# Longitudinal changes in tear cytokines and antimicrobial proteins in trachomatous disease

**DOI:** 10.1371/journal.pntd.0011689

**Published:** 2023-10-20

**Authors:** Amber Barton, Nkoyo Faal, Athumani Ramadhani, Tamsyn Derrick, Elias Mafuru, Tara Mtuy, Patrick Massae, Aiweda Malissa, Hassan Joof, Pateh Makalo, Ansumana Sillah, Anna Harte, Harry Pickering, Robin Bailey, David CW Mabey, Matthew J. Burton, Martin J. Holland

**Affiliations:** 1 Clinical Research Department, Faculty of Infectious and Tropical Diseases, London School of Hygiene and Tropical Medicine, Keppel Street, London, United Kingdom; 2 Medical Research Council Gambia at LSHTM, Atlantic Boulevard, Fajara, Banjul, The Gambia; 3 Department of Ophthalmology, Kilimanjaro Christian Medical Centre, Moshi, Tanzania; 4 National Eye Health Programme, Ministry of Health, Banjul, The Gambia; Mahidol Univ, Fac Trop Med, THAILAND

## Abstract

**Background:**

Trachoma is a neglected tropical disease caused by ocular infection with *Chlamydia trachomatis*, where repeated infections and chronic inflammation can ultimately result in scarring, trichiasis and blindness. While scarring is thought to be mediated by a dysregulated immune response, the kinetics of cytokines and antimicrobial proteins in the tear film have not yet been characterised.

**Methodology:**

Pooled tears from a Gambian cohort and Tanzanian cohort were semi-quantitatively screened using a Proteome Profiler Array to identify cytokines differentially regulated in disease. Based on this screen and previous literature, ten cytokines (CXCL1, IP-10, IFN-γ, IL-1β, IL-8, IL-10, IL-12 p40, IL-1RA, IL-1α and PDGF), lysozyme and lactoferrin were assayed in the Tanzanian cohort by multiplex cytokine assay and ELISA. Finally, CXCL1, IP-10, IL-8, lysozyme and lactoferrin were longitudinally profiled in the Gambian cohort by multiplex cytokine assay and ELISA.

**Results:**

In the Tanzanian cohort, IL-8 was significantly increased in those with clinically inapparent infection (p = 0.0086). Lysozyme, IL-10 and chemokines CXCL1 and IL-8 were increased in scarring (p = 0.016, 0.046, 0.016, and 0.037). CXCL1, IP-10, IL-8, lysozyme and lactoferrin were longitudinally profiled over the course of infection in a Gambian cohort study, with evidence of an inflammatory response both before, during and after detectable infection. CXCL1, IL-8 and IP-10 were higher in the second infection episode relative to the first (p = 0.0012, 0.044, and 0.04).

**Conclusions:**

These findings suggest that the ocular immune system responds prior to and continues to respond after detectable *C*. *trachomatis* infection, possibly due to a positive feedback loop inducing immune activation. Levels of CXC chemokines in successive infection episodes were increased, which may offer an explanation as to why repeated infections are a risk factor for scarring.

## Author summary

The neglected tropical disease trachoma is caused by repeated ocular infections with *Chlamydia trachomatis*. Progression from infection to inflammation and scarring is thought to be due to a pathogenic immune response. This study aimed to identify new associations between tear proteins and trachoma, and to study how they change over time in those with infection. We found that inflammatory chemokines and the antibacterial enzyme lysozyme were increased in scarring, while IL-8 was increased during infection. In a separate cohort study, the same inflammatory chemokines were found to increase before and after detectable infection, and were higher in repeated infections.

## Introduction

Ocular infection with *Chlamydia* (*C*.) *trachomatis* serovars A-C results in the neglected tropical disease trachoma, characterised by follicular conjunctivitis or papillary hypertrophy on the upper tarsal conjunctiva (active trachoma) [1]. However, many infections have no clinical signs [2]. Due to incomplete immunity and repeated exposure, recurrent episodes of infection and disease are common in endemic areas [3]. This chronic inflammation can result in scarring, and in severe cases progress to trichiasis and blindness. In 2021, 1.8 million people were estimated to be going blind as a result of trachoma [4] whilst 125 million lived in areas warranting trachoma control via antibiotics, facial cleanliness or environmental improvement [5].

The pathogenesis of trachoma is thought to be mediated by a dysregulated immune response [3], where infection-induced cytokines recruit tissue-damaging immune cells such as neutrophils and CD8^+^ T cells. Gene expression of multiple cytokines is raised in the conjunctiva during each stage of disease: IL-1β, TGF-β, TNF-α, IFN-γ, IL-2, IL-4, IL-12 p40 during infection; IL-1β, TGF-β, TNF-α, IL-17A, CCL18 and CXCL5 during active disease, and IL-1β, TGF-β and TNF-α in scarring [6–9]. However as cytokine production is regulated at the post-transcriptional and translational level [10], conjunctival gene expression does not necessarily reflect the levels of secreted cytokines.

Tear fluid is increasingly emerging as a non-invasive source of biomarkers for both ocular and systemic disease [11]. Produced by the lacrimal glands, tears contain over a thousand proteins, including high levels of the antimicrobial proteins lysozyme and lactoferrin [12]. Tears also contain cytokines produced by local epithelial and immune cells, with two studies previously finding TNF-α to be raised in the tears of those with trachomatous scarring [13,14].

While a number of cytokines and proteins have been identified as raised or suppressed in trachomatous disease, in the absence of longitudinal data it is difficult to discern how these changes affect disease progression. Furthermore, given the vast number of proteins in tears, there is scope for as yet undiscovered relationships between tear composition and disease. Studies to date have also focused on active disease and trachomatous scarring rather than inapparent *C*. *trachomatis* infection. We hypothesised that as yet unidentified cytokines may be associated with disease, and that longitudinal changes in tear cytokines during asymptomatic infections may relate to the downstream pathogenesis of trachoma.

We used proteome profiler arrays to semi-quantitatively screen for novel cytokines associated with different stages of trachoma. We then investigated these relationships in a Tanzanian cohort study, and tested whether tear lysozyme and lactoferrin are differentially regulated in trachoma. Finally, using tears from a second cohort study from The Gambia, we tracked changes in tear cytokines over the course of infection and disease.

## Methods

### Ethics statement

For the Gambian cohort, study procedures were approved by The Gambia Government/Medical Research Council, The Gambia Unit Joint Ethics Committee and the London School of Hygiene & Tropical Medicine Ethics Committee. For the Tanzanian cohort, study procedures were approved by the Tanzania National Institute for Medical Research, Kilimanjaro Christian Medical University College and London School of Hygiene & Tropical Medicine ethics committees. For both cohorts written informed consent was obtained from all study participants’ guardians on their behalf.

### Studies

Tear samples were collected from two longitudinal cohort studies that are detailed elsewhere [15,16]: briefly tears were included from fifteen time points in a Gambian cohort (bi-monthly over 6 months), and at two timepoints in a Tanzanian cohort (annually at months 72 and 84 post-baseline) (Table 1).

**Table 1:** Summary of the two cohorts from which tear samples were collected.

Cohort	Gambian		Tanzanian	
Age	4–15 years (entire cohort and in samples assayed)		11–19 years (entire cohort and in samples assayed)	
Participants	345		666	
	Every 2 weeks for 6 months		Every 3 months for 4 years then at months 72 and 84 post baseline	
Tear samples assayed	• 454 total • 61 participants at one timepoint, 44 at two timepoints, 23 at three, 24 at four, 6 at five, 7 at six, 6 at seven, 2 at eight, 1 at ten • 155 samples from during infection, 28 active disease and 31 scarring		• 152 total • 88 participants at one timepoint, 31 participants at two timepoints • 15 samples from during infection, 2 active disease and 54 scarring	
Grading	WHO simplified system		WHO FPC system	
	Active disease	TF	Active disease	F2 or F3
		TI		P3
	Scarring	TS	Scarring	C1, C2 or C3
		TT		
Active disease prevalence	24% (baseline timepoint)		5% (72 months post-recruitment)	
Scarring prevalence	5% (baseline timepoint)		26% (72 months post-recruitment)	

The Gambian cohort consisted of 345 children age 4–15 years recruited from nine villages. Participants were followed up every two weeks for six months [9,15]. At each visit children were clinically graded for trachoma using the WHO simplified system. Here the grades "Trachomatous inflammation—follicular (TF)" and "Trachomatous inflammation—intense (TI)" are considered "active disease", and the grades "Trachomatous scarring (TS)" and "Trachomatous trichiasis (TT)" as "scarring". Conjunctival swabs were collected from the right eye and stored in RNAlater (ThermoFisher) for subsequent *C*. *trachomatis* detection by polymerase chain reaction (PCR), as described previously [15]. Tears were collected at every timepoint by holding a sponge-tipped eye spear (Merocel, previously found to have highest recovery of tear cytokines relative to other ophthalmic sponges [17]) to the inferior conjunctival fornix for 30 seconds. The spear was then placed in a tube containing a protease inhibitor cocktail in 300μl PBS (Sigma) and stored at -70°C.

The Tanzanian cohort consisted of 666 children from three neighbouring communities, age 4–10 years at time of recruitment, and 11–19 years at the final timepoint. Participants were initially followed up every three months for four years, then at 72 and 84 months post-baseline recruitment. Tears were collected at the final two time points only. At each visit participants were clinically graded using the WHO FPC (Follicles, papillary hypertrophy, cicatriciae) system. Here a grade of F2, F3 or P3 is considered "active disease", and C1-3 considered "scarring". Conjunctival swabs were collected from the left eye and stored in RNAlater (ThermoFisher) for subsequent *C*. *trachomatis* detection by PCR [16]. Tears were collected using a standardised collection by Schirmer strips, allowing simultaneous measurement of dry eye (tear volume <15mm). Schirmer strips were stored at -70°C. After thawing, strips were placed in a 0.5ml microcentrifuge tube with a punctured base. The 0.5ml tube was placed in a 2ml microcentrifuge tube, and 20μl sterile PBS applied to the middle of the strip. Tears were finally eluted by centrifuging for 10 minutes at 15,700g in a micro-centrifuge chilled to 4°C.

### Proteome profiler array

Two proteome profiler human cytokine array kits (R&D systems, ARY005B) were used to semi-quantitively screen for cytokines of interest. For the Gambian cohort four pools were created, each containing six 100μl tear samples made up to 1.5ml using the kit array buffer provided. The pools contained: (1) samples from healthy participants, (2) samples where *C*. *trachomatis* infection was detected, (3) samples from participants with conjunctival scarring, (4) samples from participants with both *C*. *trachomatis* infection and conjunctival scarring. For the Tanzanian cohort four pools were created with each containing six 10μl tear samples made up to 1.5ml with kit array buffer. The pools contained samples from: (1) healthy participants with no history of active disease; (2) healthy participants with a history of active disease (one of more episodes of active disease preceding but not at the time of sample collection); (3) participants with conjunctival scarring and no history of active disease, and (4) conjunctival scarring with trichiasis and a history of active disease.

The assay procedure was carried out according to manufacturer’s instructions. Briefly, 15μl detection antibody cocktail was added to each pool and incubated for one hour. Meanwhile, four nitrocellulose membranes containing 36 capture antibodies (against C5a, IL-4, IL-27, CD40L, IL-5, IL-32α, G-CSF, IL-6, IP-10, GM-CSF, IL-8, CXCL11, CXCL1, IL-10, CCL2, CCL1, IL-12 p70, MIF, ICAM-1, IL-13, MIP-1, IFN-γ, IL-16, RANTES, IL-1α, IL-17, CXCL12, IL-1β, IL-17E, Serpin E1, IL-1RA, IL-18, TNF-α, IL-2, IL-21 and TREM-1) in duplicate were incubated in blocking buffer for one hour on a rocking platform shaker. Blocking buffer was aspirated from the membranes, and the detection antibody/pool mixture added. Membranes were incubated overnight at 4°C on a rocking platform shaker, then washed before incubation with Streptavidin-Horseradish Peroxidase (HRP) for 30 minutes. Membranes were once again washed and Chemi Reagent mix added. Chemiluminesence was imaged using a ChemiDoc for the Gambian cohort, and a C600 Azzure for the Tanzanian cohort. ImageJ was used to find the integrated pixel density of each spot. The mean of each duplicate spot was taken, and normalised relative to the integrated pixel density of negative control spots. As arrays were semi-quantitative and performed using pooled samples without biological replicates, no formal hypothesis-testing was carried out.

### Tear protein concentration

Total tear protein concentration was measured using a bicinchoninic acid assay (Pierce BCA Protein Assay Kit, ThermoFisher). Tears from the Tanzanian cohort were diluted 1:10 prior to protein quantitation, and protein quantitation carried out in singlicate. Protein concentration was measured in undiluted tears from the Gambian cohort in singlicate. 10μl tear samples were pipetted into a 96 well flat-bottomed plate. 200μl working reagent was added and the plate incubated for 30 minutes at 37°C. Absorbance was then read on a plate reader at 562nm. Concentrations were estimated from the standard curve using the drc package in R. For the Tanzanian cohort duplicates were averaged.

### Multiplex cytokine assays

Cytokine concentrations were measured using a MILLIPLEX Human Cytokine/Chemokine Magnetic Bead Panel (Millipore). For the Tanzanian cohort, a 10-plex (CXCL1, IL-10, IL-1α, IL-1β, IL-8, IP-10, IL-12 p40, IL1-RA, IFN-γ & PDGF-AB/BB) assay was carried out in duplicate on tears diluted 1:10 in assay buffer. For the Gambian cohort, a 3-plex (CXCL1, IL-8 and IP-10) was carried out on undiluted tears in singlicate. Samples from the same participant at different timepoints were assayed on the same plate. Concentrations falling below the range of the standard curve were set to zero (19.2% of measurements in the Tanzanian cohort; 0.1% in the Gambian cohort). One sample in the Tanzanian cohort had insufficient beads and was excluded. Where the median fluorescence intensity was too high to estimate concentration, the concentration was set to that of the highest standard for that cytokine (0.5% of measurements in the Tanzanian cohort; 1.0% in the Gambian cohort). For the Tanzanian cohort the mean of the two replicates was taken. Finally, cytokine concentration was normalised relative to total protein concentration.

### Antimicrobial protein auantification

Tear lysozyme concentrations were measured using a Human Lysozyme enzyme-linked immunosorbent assay (ELISA) Kit (abcam, ab108880), and lactoferrin concentrations using a Human Lactoferrin ELISA Kit (Immunology Consultants Laboratory, E-80LF). For the Tanzanian cohort, tears were initially diluted 1:1000,000 for lysozyme quantification, and 1:100,000 for lactoferrin quantification. Tears were assayed in duplicate according to manufacturers’ instructions (n = 152 samples for both lysozyme and lactoferrin). Any samples with an absorbance too high to estimate lysozyme concentration from the standard curve, or with an estimated lysozyme concentration > 10ng/ml diluted, were tested at a dilution of 1:10,000,000. For the Gambian cohort, tears were diluted 1:25,000 for lysozyme quantification and 1:2500 for lactoferrin quantification. Tears were assayed in singlicate according to manufacturers’ instructions (n = 454 samples for lactoferrin and n = 377 samples for lysozyme). Concentrations were estimated from the standard curve using the drc package in R. For the Tanzanian cohort duplicates were averaged.

### Data analysis

Data analysis was carried out in the R statistical environment. For comparisons between different clinical groups where multiple samples from the same participant were included, a mixed effects logistic regression model (glmer function, lme4 package) was used to adjust for age, sex and multiple observations of the same participant. For comparisons between different clinical groups where each participant was only included once, a logistic regression model adjusting for age and sex was used. As data was not normally distributed, for paired tests a Wilcoxon signed rank test was used. P values below 0.05 were considered significant. Data was visualised using the ggplot2 package. In several figures data is visualised as a log-transformed fold change relative to the first infection time point. This timepoint was chosen as it was tested in more participants than any other timepoint, and allows the direction of change to be identified for each participant.

### Transcriptomic Re-analysis

Transcriptomic datasets GSE20436, GSE20430 [18], GSE114556 [19], GSE105149 [20], GSE180238 [21] and GSE180027 [22] were downloaded from the NCBI Gene Expression Omnibus using the Bioconductor R package GEOquery. If not already, datasets were log-transformed, and quantile-normalised using the preprocessCore R package. Rows were collapsed to genes using the WGCNA package, taking the probe with the maximum mean expression as representative [23]. Differential expression analysis between conditions was carried out using the limma package. To compare gene expression between tissues, non-log transformed average expression was normalised relative to *ACTB*.

## Results

### Tear inflammatory cytokines are raised in scarring in a Semi-quantitative screen of pooled tear samples

**Fig 1:**
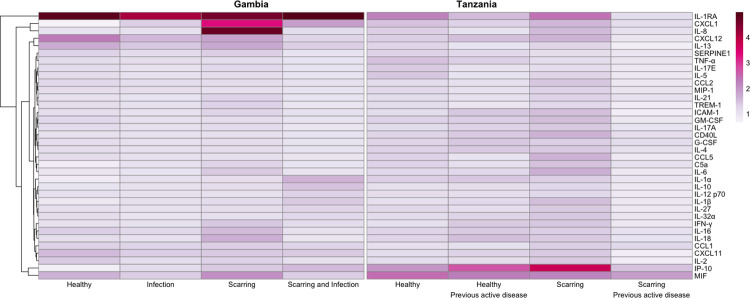
Mean integrated pixel density of each cytokine relative to negative control spots in each of eight pooled tear samples. Clinical status of each pool is indicated on the x axis. Cytokines are clustered by euclidean distance. As arrays were semi-quantitative and performed using pooled samples without biological replicates, no formal hypothesis-testing was carried out.

In the initial screen using pooled tear samples and semi-quantitative arrays, relative to the healthy control pools CXCL1, IP-10, IFN-γ, IL-1β, IL-8, IL-10, IL-12 and IL-27 were higher in Gambian participants with scarring and in Tanzanian participants with scarring but no active disease history (Fig 1). Relative to healthy controls, IL-1α was higher in Gambian participants with scarring, but not Tanzanian participants with scarring. IL-1RA was constitutively present in tears at high levels. Based on this screen CXCL1, IP-10, IFN-γ, IL-1β, IL-8, IL-10, IL-12 p40, IL1-RA and IL-1α were selected for follow up multiplex cytokine assays on individual Tanzanian samples. IL-27 was not selected for further follow up due to lack of differential expression in microarray data [18]. PDGF-AB/BB, while not included in the semi-quantitative array, was selected for inclusion in the multiplex cytokine assay since it has previously been associated with conjunctival damage [24]. Lactoferrin and lysozyme concentrations were also assayed by ELISA as these had been suggested as markers of tear film health [25].

### Tear lysozyme and CXC chemokines are raised in scarring in a Tanzanian Cohort study

**Fig 2:**
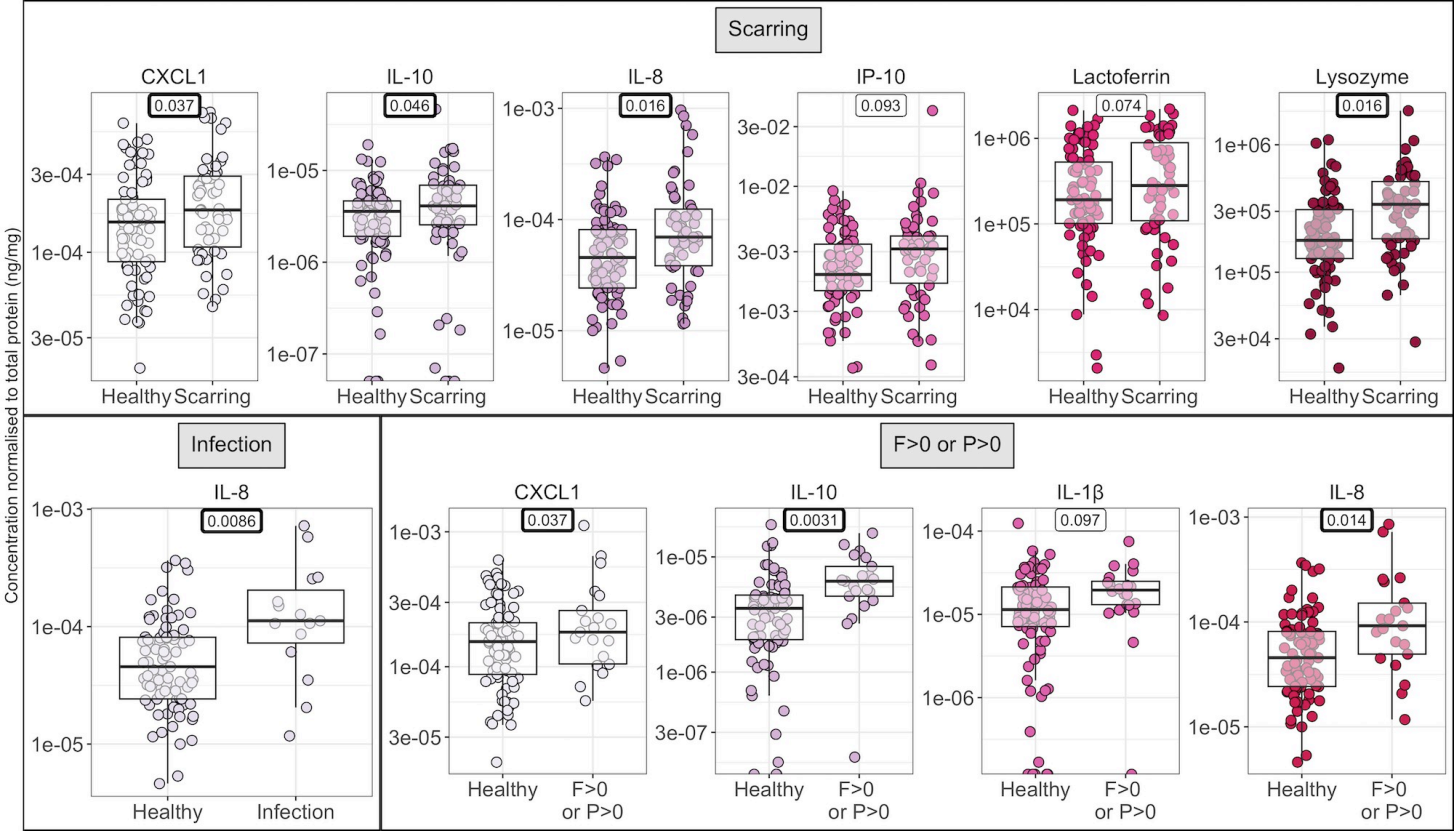
Protein concentration for participants in different infection and clinical categories in the Tanzanian Cohort study. Comparisons with a p value < 0.1 for a mixed-effects logistic regression adjusting for age, sex and participant is shown. Comparisons with a p value < 0.05 are highlighted in bold. Each point represents a sample, coloured by cytokine/antimicrobial protein. Box plots indicate median and interquartile range (IQR), with whiskers extending to the most extreme values within 1.5 x IQR. Tear samples collected at both at 72 and 84 months post-recruitment were included.

As tears were collected at two timepoints in the Tanzanian Cohort study (72 and 84 months post-recruitment), with some but not complete overlap in participants at each timepoint, a mixed effects logistic regression model was used to compare each group to healthy controls, adjusting for age, sex and multiple observations of the same participant. Compared with healthy controls (no infection, FPC clinical grades all zero), tear samples from Tanzanian cohort participants infected with *C*. *trachomatis* had higher levels of IL-8 (p = 0.0086, mixed effects logistic regression; Fig 2). In samples from those with scarring, lysozyme, IL-10 and CXC chemokines IL-8 and CXCL1 were significantly increased (p = 0.016, 0.046, 0.016, and 0.037 respectively, mixed effects logistic regression) while IP-10 and lactoferrin fell just short of significance (p = 0.093 and 0.074 respectively). While there were too few participants with active trachoma (clinical grades F > 1, P > 2, n = 2) to detect changes in active disease, IL-10, IL-8 and CXCL1 were significantly increased in those with clinical grades F > 0 or P > 0 (p = 0.0031, 0.014, 0.037 respectively, logistic regression). Consistently, re-analysis of a gene expression array performed in a cross-sectional Gambian study independent of the samples assayed in this study [18] identified the genes encoding IL-10, IL-8 and CXCL1 as being upregulated (p < 0.05, linear modelling) during active disease (Table A in S1 Text).

### CXCL1 and lactoferrin are raised in Gambian participants with *C*. *trachomatis* infection

CXCL1, IP-10, IL-8, lysozyme and lactoferrin were selected for further profiling in a Gambian cohort study with more closely spaced and a greater number of timepoints available. Samples were selected to investigate the kinetics of infection and scarring (Table 2). Recovery of CXCL1, IP-10 and IL-8 from sponge-tipped eye spears in the Gambian cohort study was higher than from Schirmer strips in the Tanzanian study, while lysozyme and lactoferrin were lower (Fig A in S1 Text).

**Table 2:** Tear samples selected from the Gambian cohort study to investigate the kinetics of infection and scarring.

Subgroup	Timepoints assayed	Number of samples
Healthy throughout study (no positive PCRs or clinical signs throughout study)	One timepoint to act as control	43
Infection	First timepoint of infection	128
	Further timepoints during infection	27
	Timepoints leading up to infection	85
	Timepoints after infection	154
Scarring throughout the study period	Timepoint during scarring	2
Developed scarring during the study	Timepoints prior to scarring onset	35
	Timepoints after development of scarring	29
Active disease	Timepoints during active disease	28

**Fig 3:**
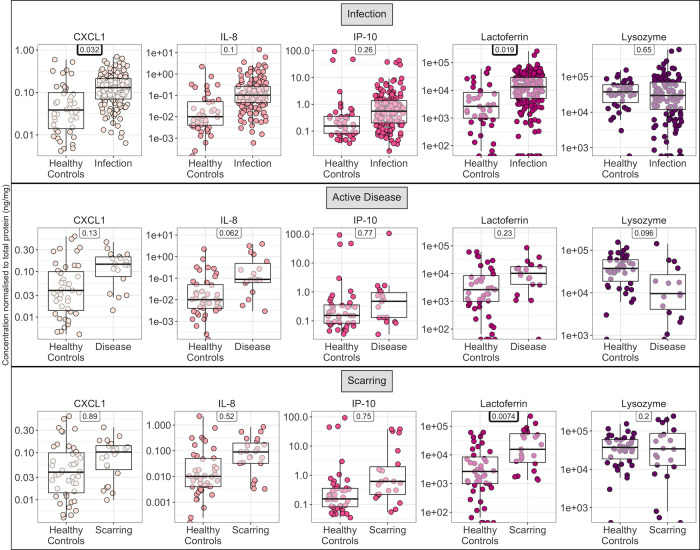
Protein concentration for participants in different infection and clinical categories in the Gambian Cohort study, including samples from all time points. Samples are categorised by whether they came from a) healthy controls, who did not develop any infection or disease over the course of the study or from b) participants at a time point at which they had infection/disease/scarring. P values for a mixed-effects logistic regression adjusting for age, sex and participant are indicated, with those < 0.05 highlighted in bold. Each point represents a sample, coloured by cytokine/antimicrobial protein. Box plots indicate median and IQR with whiskers extending to the most extreme values within 1.5 x IQR.

Analysing all timepoints together in an unpaired analysis, there was a marked trend towards higher CXCL1, IL-8, IP-10 and lactoferrin during infection. However, after adjusting for age, sex and repeated sampling of the same participant, only CXCL1 and lactoferrin reached significance (p = 0.032 and 0.019 respectively, mixed effects logistic regression; Fig 3). Active disease and scarring followed a similar pattern, with only an increase in lactoferrin during scarring reaching significance (p = 0.0074). For lysozyme on the other hand, there was a non-significant decrease in active disease (p = 0.096, logistic regression). Re-analysis of microarray data suggested that at the gene expression level, lysozyme is upregulated in the conjunctiva during active disease (p = 0.0001, log_2_(fold change) = 0.5, linear modelling) while expression in the lacrimal gland is halved during inflammation (GSE105149, p = 0.1, log_2_(fold change) = -1.0), suggesting the drop is likely mediated by reduced lacrimal gland secretion. In contrast to the Tanzanian Cohort study, lysozyme was unchanged in scarring (p = 0.2).

### CXC inflammatory chemokines increase prior to detectable infection, peak four weeks after infection and are increased in successive infection episodes

**Fig 4:**
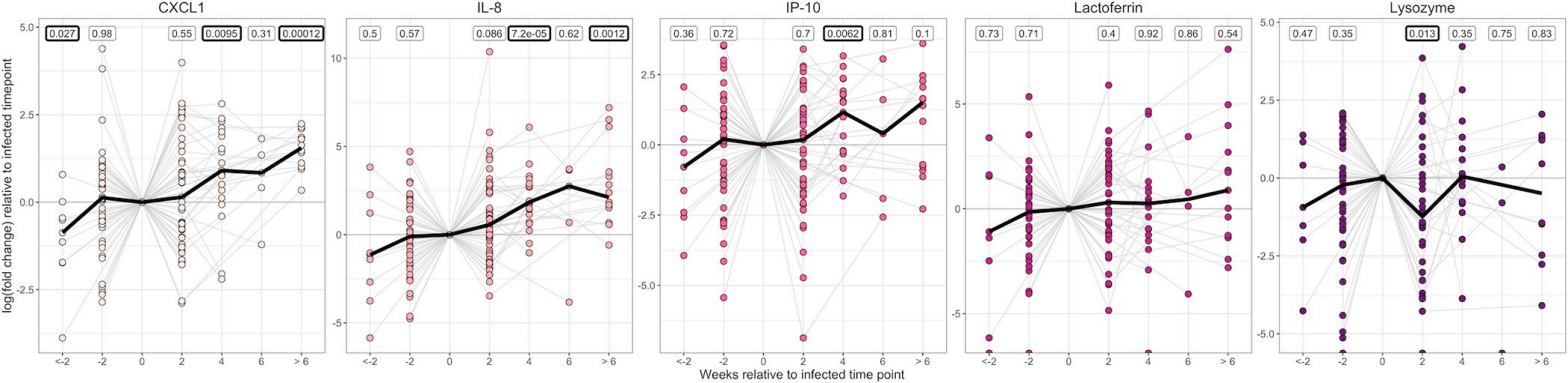
Change in normalised protein concentration relative to the infected time point for each participant in the Gambian Cohort study. Time points are separated by two weeks, and samples were limited to the first infection episode only. P values for a paired Wilcoxon signed-rank test comparing the normalised concentration at each time point relative to time of infection are shown, with p values < 0.05 highlighted in bold. Each point represents a sample, coloured by cytokine/antimicrobial protein. Grey lines show the log_2_(fold change) in concentration over time for each participant, while the black thick line shows the median log_2_(fold change).

**Fig 5:**
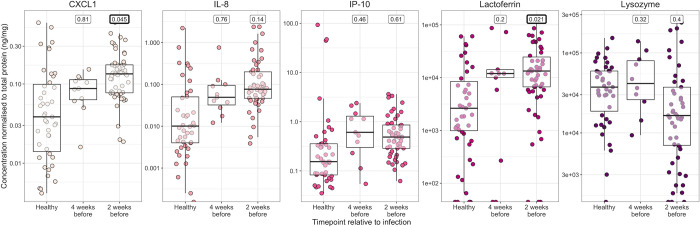
Normalised protein concentrations prior to infection, compared with healthy controls. Protein concentrations normalised to total protein, in healthy control participants (individuals with no PCR positive infections or clinical signs throughout the entire study) compared with four weeks and two weeks prior to first detectable infection in individuals who did develop infection. Only the first episode of infection was analysed. P values for a logistic regression model comparing each timepoint with healthy controls, adjusting for age and sex, is shown. P values < 0.05 are highlighted in bold. Each point represents a sample, coloured by cytokine/antimicrobial protein. Box plots indicate median and IQR, with whiskers extending to the most extreme values within 1.5 x IQR.

Focusing only on the first episode of infection in the Gambian Cohort study, inflammatory CXC cytokines were raised four weeks after infection (p = 0.0095, 7.2 x 10^−5^ and 0.0062 for CXCL1, IL-8 and IP-10 respectively, using a paired Wilcoxon signed-rank test to compare the normalised concentration four weeks post-infection relative to time of infection; Fig 4). Lysozyme however significantly dropped two weeks after infection (p = 0.013, paired Wilcoxon signed-rank test) while lactoferrin did not change. Compared with healthy controls who had no clinical signs or positive PCRs throughout the course of the study, CXCL1 and lactoferrin were raised two weeks before *C*. *trachomatis* infection was detected (p = 0.045 and 0.021, logistic regression model adjusting for age and sex; Fig 5).

A transcriptomic re-analysis was carried out to identify which tissues may be responsible for these changes (Table B in S1 Text). Lysozyme was highly expressed by the lacrimal gland, but also expressed by phagocytes and epithelial cells. CXC chemokines were expressed by epithelial and/or immune cells rather than the lacrimal gland (Table B in S1 Text), and were upregulated in response to in vitro *C*. *trachomatis* infection or LPS stimulation (Table C in S1 Text).

In participants with sustained infections lasting two or more weeks, chemokines and antimicrobial proteins remained constant during infection (Fig B in S1 Text). There were no significant changes in the timepoints leading up to active disease or scarring (Figs C and D in S1 Text).

**Fig 6:**
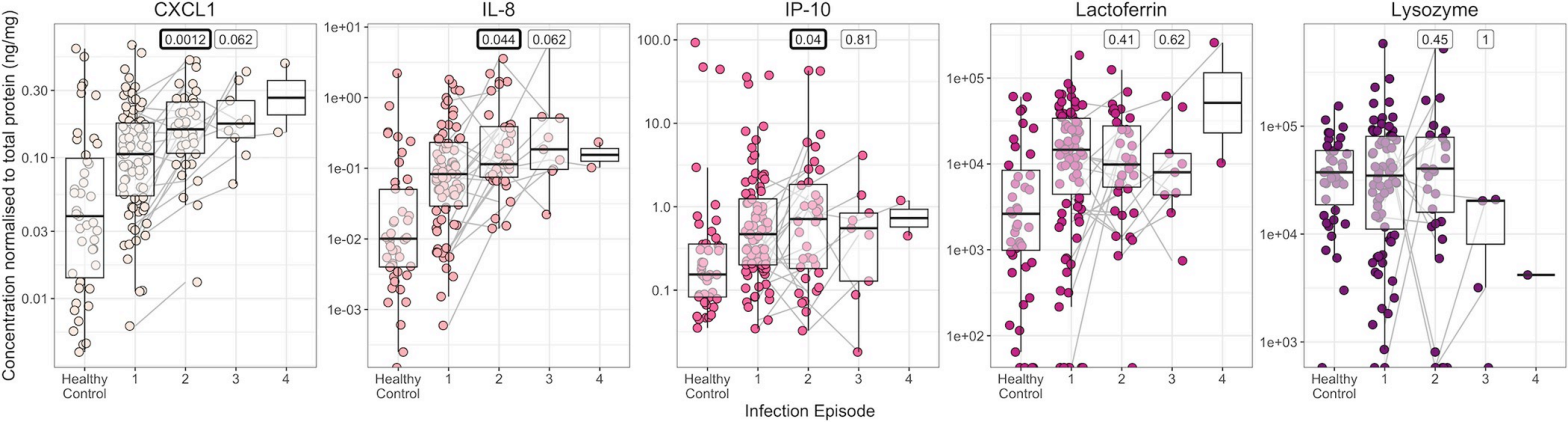
Concentration of proteins normalised to total tear protein at the first timepoint of each infection episode. Healthy controls are shown for comparison. P values for a paired Wilcoxon signed-rank test comparing episodes 2 and 3 with episode 1 are shown, with p values < 0.05 highlighted in bold. Each point represents a sample, coloured by cytokine/antimicrobial protein. Box plots indicate median and IQR, with whiskers extending to the most extreme values within 1.5 x IQR.

In participants with successive infections, the second and third infection episodes were compared to the first infection episode (first time point of each episode only). CXCL1, IL-8 and IP-10 were raised in the second infection episode relative to the first (p = 0.0012, 0.044, and 0.04, paired Wilcoxon signed-rank test, n = 20; Fig 6).

## Discussion

### Kinetics of tear cytokine responses to *C*. *trachomatis* infection

These data represent the first report of cytokines and antimicrobial proteins longitudinally profiled in the tears of human participants over the course of ocular *C*. *trachomatis* infection and trachomatous disease. We found that CXC cytokines IL-8, IP-10 and CXCL1, and antimicrobial protein lactoferrin, were raised either during or following clinically inapparent infection. Consistently, following exposure to *C*. *trachomatis* the genes encoding IL-8 and IP-10 are upregulated by neutrophils [21], IL-8 and CXCL1 by macrophages [26], and CXCL1 by epithelial cells [19]. IL-8, IP-10 and CXCL1 are all transcriptionally regulated by NF-κB [27], suggesting that cytokine expression is likely induced by pattern recognition receptors: either directly by innate recognition of *C*. *trachomatis* [28,29], or indirectly in response to *C*. *trachomatis*-mediated cell damage [30].

**Fig 7:**
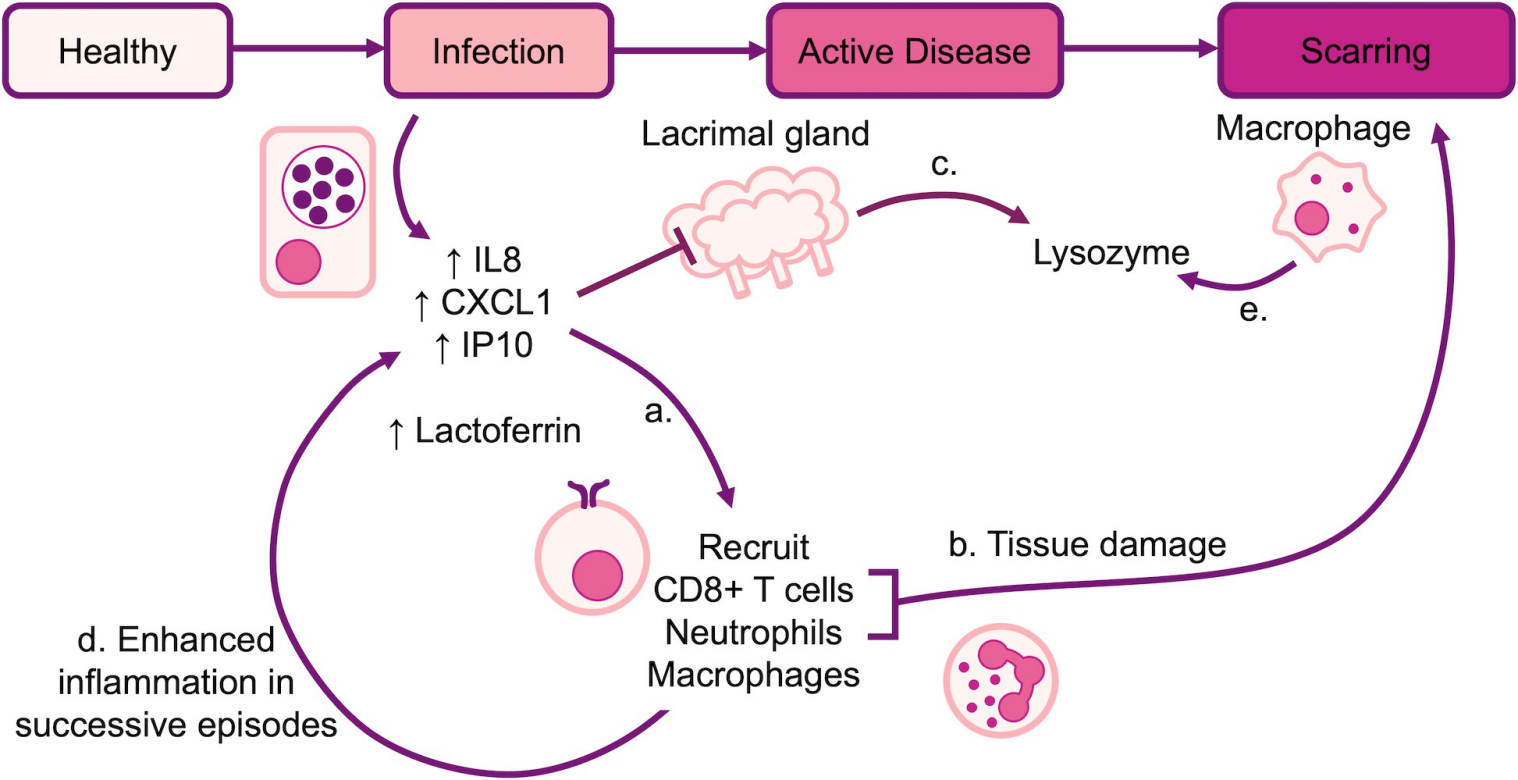
A model for trachoma pathogenesis based on study findings. a. Infection results in activation of pattern recognition receptors and therefore an increase in CXC chemokines, resulting in recruitment of T cells, Neutrophils and Macrophages b. CD8^+^ T cells and neutrophils can mediate tissue damage, resulting in trachomatous scarring c. Inflammatory cytokines suppress lacrimal gland secretion, decreasing lysozyme tear concentration d. Immune cells recruited to the conjunctiva and/or immune memory result in enhanced inflammation in successive infection episodes e. At later stages of scarring lysozyme is raised, possibly due to secretion by recruited macrophages.

Whereas lactoferrin is anti-inflammatory and prevents *C*. *trachomatis* invasion in vitro [31], CXC cytokines are a double edged-sword, recruiting neutrophils and T cells to sites of infection [32] (Fig 7A). While CD4^+^ T cell responses against *C*. *trachomatis* are necessary for resolution of infection, CD8^+^ T cells and neutrophils may mediate the tissue damage seen in trachomatous scarring (Fig 7B) [3,33]. Animal models suggest that while CD8^+^ T cells cause pathology through secretion of TNF-α [34,35], neutrophils may directly damage tissue through production of reactive oxygen species and matrix metalloproteinases [3,36]. In support of neutrophil recruitment playing a role in pathogenesis, a genetic variant resulting in lower levels of IL-8 increases resistance to trachomatous scarring [37].

Inflammatory cytokines can also inhibit lacrimal gland tear secretion [25,38]. This may explain why the antibacterial enzyme lysozyme, which is secreted by both the lacrimal gland and phagocytes in the conjunctiva, is suppressed two weeks after infection (p = 0.013) with a non-significant drop during active disease (p = 0.096) (Fig 7C). While lysozyme has not previously been measured in tears in relation to trachoma, a fall in levels has previously been observed in upper respiratory tract infections and dry eye disorders [25,39].

Interestingly, lactoferrin and CXCL1 were significantly higher than healthy controls two weeks prior to detectable infection (p = 0.045 and 0.021), suggesting an immune response is triggered well before *C*. *trachomatis* reaches a high enough bacterial load to be detected by 16s rRNA PCR. Furthermore, CXCL1, IP-10 and IL-8 peaked at around four weeks post-infection, even though by this time *C*. *trachomatis* was no longer detectable by PCR. One possibility is that after an infection has been resolved or suppressed, a positive feedback loop continues to propagate an inflammatory immune response [40]. Another possibility is that live *C*. *trachomatis* suppresses inflammatory responses during infection, and this suppression wanes once the bacteria are killed. For example, in vitro IP-10 levels inversely correlate with bacterial burden, suggesting active suppression by *C*. *trachomatis* [41]. It is also possible that while not detectable in the conjunctiva, *C*. *trachomatis* is present at a sub-detectable level, or persisting in other ocular tissues or the lacrimal gland. Finally, it may be that other bacteria contribute to inflammation following resolution of infection: for example, it has previously been found that *Streptococcus pneumoniae* and *Haemophilus influenzae* are more commonly cultured from those with active trachoma than healthy controls [42,43].

We also found that CXC chemokine responses were higher in the second infection episode relative to the first episode. This could explain why repeated infection episodes increase the likelihood of scarring and may be due to several mechanisms. Firstly, it may be that the immune cells recruited to the conjunctiva during the first episode are still present, and therefore mount a stronger inflammatory response in subsequent episodes (Fig 7D). Secondly, activation of memory T cells could result in an enhanced secondary immune response. Finally, epigenetic changes in immune cells could result in a “trained” innate immune response during subsequent infection episodes [44].

Our results contrasted to others in that unlike previous gene expression analyses, which found that infection induced IFN-γ in the conjunctiva [6,8,9,15], our assay was not sufficiently sensitive to consistently detect IFN-γ in the tears. Furthermore, we did not detect a significant change in IL-12 or IL-1β during infection [6,8,9].

### Cytokines in active disease and scarring

No significant (p < 0.05) changes in cytokines were detected during active disease, likely due to low numbers of samples in the Tanzanian cohort, and selection of samples to focus on infection in the Gambian cohort. However, in the Tanzanian cohort IL-10, IL-8 and CXCL1 were raised in those with clinical grades F > 0 or P > 0 (p < 0.05), while in the Gambian cohort there was a non-significant increase in IL-8 and CXCL1 in active disease (p < 0.15). This is consistent with transcriptomic arrays finding an increase in conjunctival IL-10, IL-8 and CXCL1 mRNA [18], quantitative PCR finding an increase in conjunctival IL-10 mRNA [8], and cytokine assays finding an increase in tear IL-8 and IL-10 [14] during active disease. A variety of cells could be responsible for secreting these cytokines: for example, monocytes, dendritic cells and B cells have each been identified as potential sources of IL-10 following *Chlamydia* infection [45–47]. Furthermore, conjunctival *FOXP3* expression is increased in active trachoma, suggesting that regulatory T cells might be present and contribute to tear IL-10 [15].

In the Tanzanian cohort CXCL1, IL-10 and IL-8 were significantly increased in scarring (p < 0.05). CXCL1 and IL-8 were also higher in Gambian cohort samples with scarring, but this difference was not significant after adjusting for repeated measures. These findings are in contrast to Skwor et al. 2008 [14], which found that IL12 and IL-1β were elevated in scarring, while IL-10 and IL-8 were unchanged. However, both suggest the ocular mucosa remains in an inflammatory state during scarring. In the Tanzanian study lysozyme was significantly increased (p = 0.016) in scarring, whereas in the Gambian cohort study lysozyme was unchanged. This difference could be due to the children in the Tanzanian study being older, and therefore at a later scarring stage where phagocyte secretion contributes more to tear lysozyme than the lacrimal gland (Fig 7E). Lactoferrin was significantly raised in scarring in the Gambian cohort (p = 0.0074) and was raised but fell short of significance in the Tanzanian cohort (p = 0.074). While this is likely due to sustained inflammation, it is not yet known whether lactoferrin plays any causative role in progressing or attenuating scarring trachoma.

### Limitations

This study was limited in that the two cohort studies assayed here are not directly comparable. Firstly, the Gambian cohort were younger, and therefore had a higher prevalence of infection and lower prevalence of scarring. Furthermore, while in the Tanzanian cohort infection was detected by a multiplex DNA PCR targeting genes omcB and pORF2, in the Gambian cohort infection was detected by a Chlamydial 16s rRNA PCR. Although both methods have >90% specificity when compared to commercial kits, the multiplex DNA PCR has 90% sensitivity and the 16s RNA PCR 75% sensitivity [48,49]. Tears were also collected by two different methods: surgical eye sponges in the Gambian study, and Schirmer strips in the Tanzanian study, resulting in differences in cytokine and antimicrobial protein yield (Fig A in S1 Text). Further impact from method of collection, time in storage and additives cannot be completely excluded. However, to avoid protein degradation, all samples were collected on ice in the field and stored at -70°C; Gambian samples were stored with the addition of a cocktail of protease inhibitors for long term storage; and samples selected had not been subjected to freeze-thaw prior to use on the array profiler and multiplex cytokine assays.

### Conclusions

Through a semi-quantitative screen we identified ten cytokines of interest for further follow up. IL-8 was raised in *C*. *trachomatis* infection, and multiple cytokines and lysozyme raised in conjunctival scarring. CXCL1, IP-10, IL-8, lysozyme and lactoferrin were longitudinally profiled over the course of infection in a separate cohort, with evidence of an inflammatory response both before and after detectable infection. Increased inflammation was observed in later infection episodes. This could have implications in vaccine development, suggesting that natural immune responses contribute to rather than protect against pathogenesis.

## Supporting information

S1 Text **Fig A. Comparison of protein recovery from Schirmer strips in the Tanzanian cohort study with recovery from sponge-tipped eye spears in the Gambian cohort study.** Both analyte concentration (ng/ml) and analyte concentration normalised to total protein (ng/mg total protein) are shown. Points each represent one sample, coloured by whether they represent a healthy control or other sample. Box plots indicate median and IQR, with whiskers extending to the most extreme values within 1.5 x IQR.**Fig B. Protein concentration at the first infected time point, two weeks after infection and four weeks after infection in participants with sustained infections.** Paired tests were carried out on time points from the same infection episode only. P values for a paired Wilcoxon signed-rank test comparing the normalised concentration at each time point relative to the first time point of infection are shown. Each point represents a sample. Grey lines show the change in concentration over time for each participant and infection episode, while the black thick line shows the change in the median. **Fig C. Change in normalised protein concentration relative to the first scarring time point for each participant.** Samples were limited to the first scarring episode only. P values for a paired Wilcoxon signed-rank test comparing the normalised concentration at each time point relative to time of incident scarring are shown. Each point represents a sample, coloured by cytokine/antimicrobial protein. Grey lines show the log_2_(fold change) in concentration over time for each participant, while the black thick line shows the median log_2_(fold change). **Fig D. Change in normalised protein concentration relative to the first active disease time point for each participant.** Samples were limited to those without any scarring or infection. P values for a paired Wilcoxon signed-rank test comparing the normalised concentration at each time point relative to time of active disease onset are shown. Each point represents a sample, coloured by cytokine/antimicrobial protein. Grey lines show the log_2_(fold change) in concentration over time for each participant, while the black thick line shows the median log_2_(fold change).**Table A. Differential expression of *IL10*, *IL1B*, *IL8* and *CXCL1* in the conjunctiva in active trachomatous disease relative to healthy controls in datasets GSE20436 and GSE20430.** Samples were taken from a Gambian cross-sectional case-control study. Gene expression was quantile normalised and probes were collapsed to genes, taking the probe with the maximum mean as being representative. Differential expression analysis was carried out using the Bioconductor R package limma. **Table B.Mean expression of *CXCL1*, *IL8*, *CXCL10 (IP10)*, *LYZ* and *LTF* relative to *ACTB*, in epithelial cells, neutrophils, monocytes, macrophages, activated macrophages and the lacrimal gland.** Datasets GSE114556, GSE105149, GSE180238 and GSE180027 were downloaded, quantile normalised and probes were collapsed to genes, taking the probe with the maximum mean as being representative. The mean expression of each gene (non-log transformed) was normalised relative to *ACTB* expression.**Table C.Differential expression of *CXCL1*, *IL8*, *CXCL10 (IP10)*, *LYZ* and *LTF* following in vitro exposure of epithelial cells, neutrophils or macrophages to either *C*. *trachomatis* or LPS.** Datasets GSE114556, GSE180238 and GSE180027 were downloaded, quantile normalised and probes were collapsed to genes, taking the probe with the maximum mean as being representative. Differential expression analysis was carried out using the Bioconductor R package limma.
(DOCX)

S1 Dataset Data generated by this study. Integrated pixel densities normalised to negative control spots for the proteome profiler array screen; target analyte and total protein concentrations for each sample in the Tanzanian cohort study; and target analyte and total protein concentrations for each sample in the Gambian cohort study.
(XLSX)
