# Supplementary material for: Longitudinal changes in tear cytokines and antimicrobial proteins in trachomatous disease
Source: PLoS Negl Trop Dis. 2023 Oct 20;17(10):e0011689. doi: 10.1371/journal.pntd.0011689 (PMC10619880; doi:10.1371/journal.pntd.0011689)
Supplement: S1 Text — Fig A. Comparison of protein recovery from Schirmer strips in the Tanzanian cohort study with recovery from sponge-tipped eye spears in the Gambian cohort study. Both analyte concentration (ng/ml) and analyte concentration normalised to total protein (ng/mg total protein) are shown. Points each represent one sample, coloured by whether they represent a healthy control or other sample. Box plots indicate median and IQR, with whiskers extending to the most extreme values within 1.5 x IQR.Fig B. Protein concentration at the first infected time point, two weeks after infection and four weeks after infection in participants with sustained infections. Paired tests were carried out on time points from the same infection episode only. P values for a paired Wilcoxon signed-rank test comparing the normalised concentration at each time point relative to the first time point of infection are shown. Each point represents a sample. Grey lines show the change in concentration over time for each participant and infection episode, while the black thick line shows the change in the median. Fig C. Change in normalised protein concentration relative to the first scarring time point for each participant. Samples were limited to the first scarring episode only. P values for a paired Wilcoxon signed-rank test comparing the normalised concentration at each time point relative to time of incident scarring are shown. Each point represents a sample, coloured by cytokine/antimicrobial protein. Grey lines show the log2(fold change) in concentration over time for each participant, while the black thick line shows the median log2(fold change). Fig D. Change in normalised protein concentration relative to the first active disease time point for each participant. Samples were limited to those without any scarring or infection. P values for a paired Wilcoxon signed-rank test comparing the normalised concentration at each time point relative to time of active disease onset are shown. Each point repre [file pntd.0011689.s001.docx]

**Fig A: Comparison of protein recovery from Schirmer strips in the Tanzanian cohort study with recovery from sponge-tipped eye spears in the Gambian cohort study.** Both analyte concentration (ng/ml) and analyte concentration normalised to total protein (ng/mg total protein) are shown. Points each represent one sample, coloured by whether they represent a healthy control or other sample. Box plots indicate median and IQR, with whiskers extending to the most extreme values within 1.5 x IQR.

**
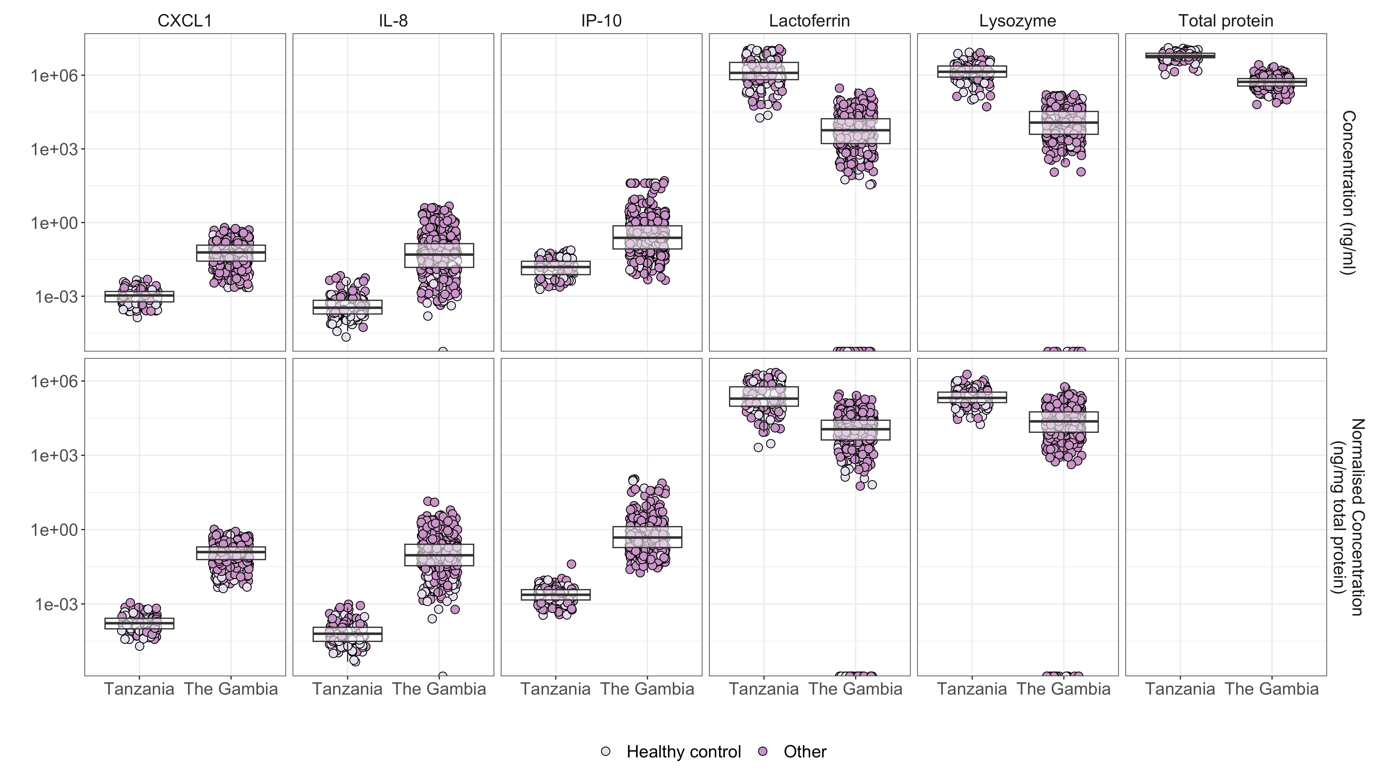
**

**Fig B: Protein concentration at the first infected time point, two weeks after infection and four weeks after infection in participants with sustained infections.** Paired tests were carried out on time points from the same infection episode only. P values for a paired Wilcoxon signed-rank test comparing the normalised concentration at each time point relative to the first time point of infection are shown. Each point represents a sample. Grey lines show the change in concentration over time for each participant and infection episode, while the black thick line shows the change in the median.


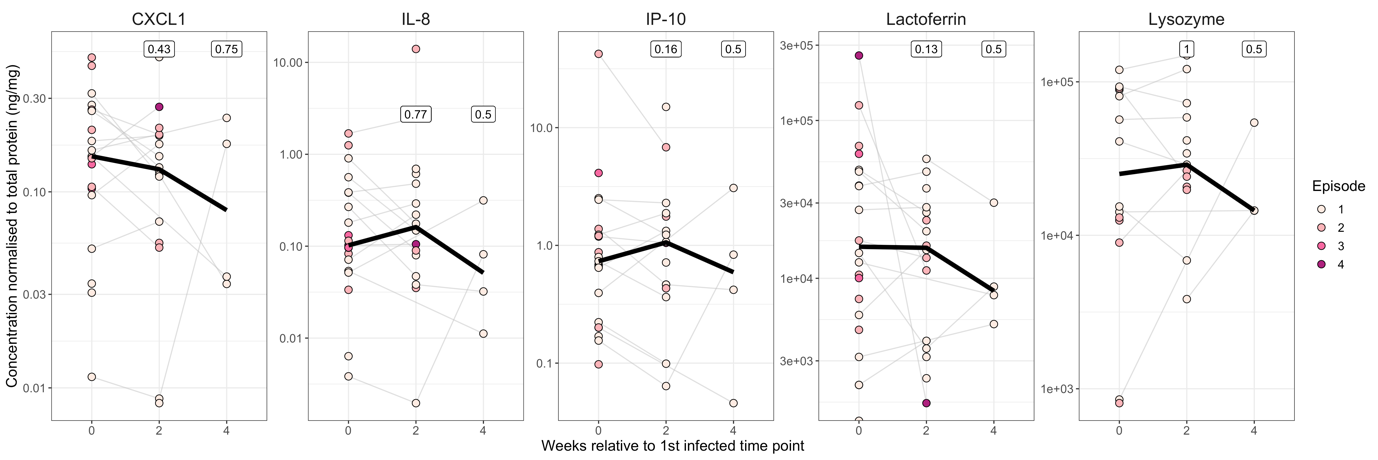


**Fig C: Change in normalised protein concentration relative to the first scarring time point for each participant.** Samples were limited to the first scarring episode only. P values for a paired Wilcoxon signed-rank test comparing the normalised concentration at each time point relative to time of incident scarring are shown. Each point represents a sample, coloured by cytokine/antimicrobial protein. Grey lines show the log_2_(fold change) in concentration over time for each participant, while the black thick line shows the median log_2_(fold change).


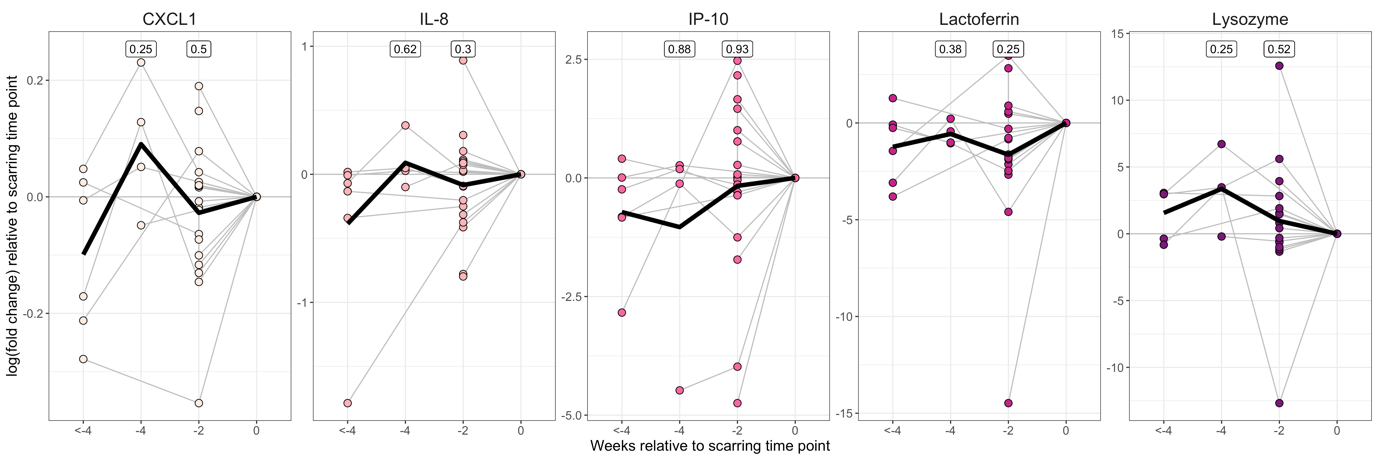


**Fig D: Change in normalised protein concentration relative to the first active disease time point for each participant.** Samples were limited to those without any scarring or infection. P values for a paired Wilcoxon signed-rank test comparing the normalised concentration at each time point relative to time of active disease onset are shown. Each point represents a sample, coloured by cytokine/antimicrobial protein. Grey lines show the log_2_(fold change) in concentration over time for each participant, while the black thick line shows the median log_2_(fold change).


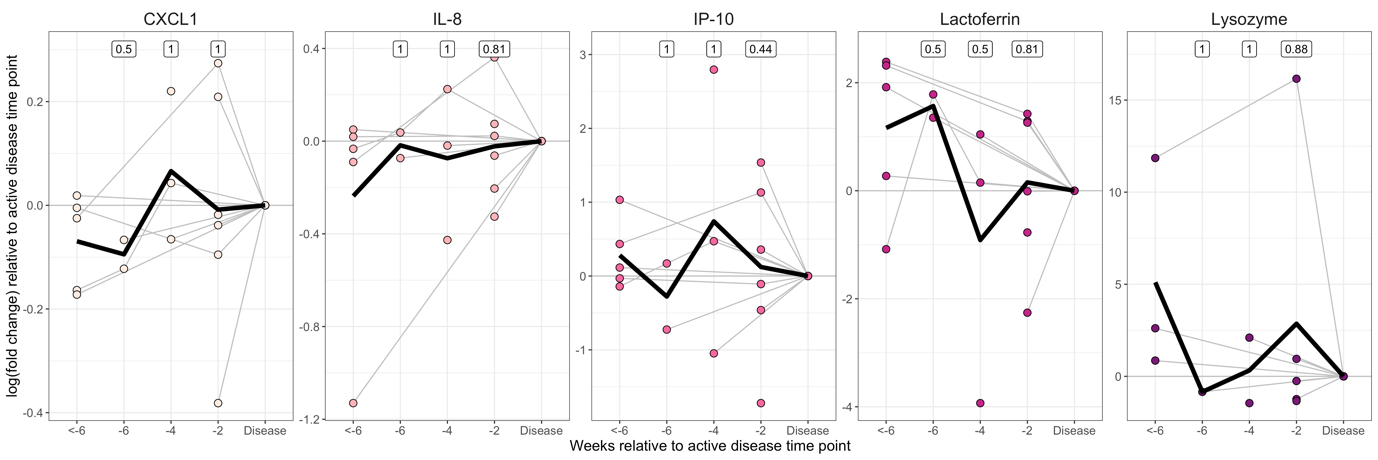


**Table A: Differential expression of *IL10, IL1B, IL8* and *CXCL1* in the conjunctiva in active trachomatous disease relative to healthy controls in datasets GSE20436 and GSE20430.** Samples were taken from a Gambian cross-sectional case-control study. Gene expression was quantile normalised and probes were collapsed to genes, taking the probe with the maximum mean as being representative. Differential expression analysis was carried out using the Bioconductor R package limma.

| **Gene** | **Dataset** | **log_2_(fold change)** | **P value** |
| --- | --- | --- | --- |
| *CXCL1* | GSE20430 | 1.14811 | 1.10x10^-4^ |
| *IL8* | GSE20430 | 0.993479 | 5.62x10^-4^ |
| *IL10* | GSE20430 | 1.221782 | 1.02x10^-3^ |
| *CXCL1* | GSE20436 | 0.90071 | 2.26x10-2 |
| *IL8* | GSE20436 | 1.161928 | 4.68x10^-2^ |
| *IL10* | GSE20436 | 0.026937 | 0.228 |

**Table B: Mean expression of *CXCL1, IL8, CXCL10 (IP10), LYZ* and *LTF* relative to *ACTB,* in epithelial cells, neutrophils, monocytes, macrophages, activated macrophages and the lacrimal gland.** Datasets GSE114556, GSE105149, GSE180238 and GSE180027 were downloaded, quantile normalised and probes were collapsed to genes, taking the probe with the maximum mean as being representative. The mean expression of each gene (non-log transformed) was normalised relative to *ACTB* expression.

| **Dataset** | **Gene** | **Tissue** | **Mean Expression (Relative to ACTB)** |
| --- | --- | --- | --- |
| GSE114556 | *CXCL1* | Epithelial (HeLa) | 0.91 |
| GSE105149 |  | Lacrimal gland | 0.00 |
| GSE180238 |  | Neutrophils (HL60) | 0.00 |
| GSE180027 |  | Monocytes | 0.00 |
| GSE180027 |  | Macrophages | 0.00 |
| GSE180027 |  | Activated macrophages (LPS-stimulated) | 0.04 |
| GSE114556 | *IL8* | Epithelial (HeLa) | 0.51 |
| GSE105149 |  | Lacrimal gland | 0.03 |
| GSE180238 |  | Neutrophils (HL60) | 0.75 |
| GSE180027 |  | Monocytes | 0.00 |
| GSE180027 |  | Macrophages | 0.00 |
| GSE180027 |  | Activated macrophages (LPS-stimulated) | 0.92 |
| GSE114556 | *CXCL10 (IP10)* | Epithelial (HeLa) | 0.27 |
| GSE105149 |  | Lacrimal gland | 0.02 |
| GSE180238 |  | Neutrophils (HL60) | 0.04 |
| GSE180027 |  | Monocytes | 0.00 |
| GSE180027 |  | Macrophages | 0.00 |
| GSE180027 |  | Activated macrophages (LPS-stimulated) | 0.01 |
| GSE114556 | *LTF* | Epithelial (HeLa) | 0.25 |
| GSE105149 |  | Lacrimal gland | 2.59 |
| GSE180238 |  | Neutrophils (HL60) | 0.00 |
| GSE180027 |  | Monocytes | 0.00 |
| GSE180027 |  | Macrophages | 0.00 |
| GSE180027 |  | Activated macrophages (LPS-stimulated) | 0.00 |
| GSE114556 | *LYZ* | Epithelial (HeLa) | 0.27 |
| GSE105149 |  | Lacrimal gland | 1.82 |
| GSE180238 |  | Neutrophils (HL60) | 1.00 |
| GSE180027 |  | Monocytes | 0.27 |
| GSE180027 |  | Macrophages | 0.07 |
| GSE180027 |  | Activated macrophages (LPS-stimulated) | 0.13 |

**Table C: Differential expression of *CXCL1, IL8, CXCL10 (IP10), LYZ* and *LTF* following in vitro exposure of epithelial cells, neutrophils or macrophages to either *C. trachomatis* or LPS.** Datasets GSE114556, GSE180238 and GSE180027 were downloaded, quantile normalised and probes were collapsed to genes, taking the probe with the maximum mean as being representative. Differential expression analysis was carried out using the Bioconductor R package limma.

| **Dataset** | **Tissue** | **Stimulus** | **Gene** | **log_2_(fold change)** | **P value** |
| --- | --- | --- | --- | --- | --- |
| GSE114556 | Epithelial (HeLa) | *C. trachomatis* | *CXCL1* | 0.512196 | 4.04x10^-2^ |
|  |  |  | *CXCL10 (IP10)* | 0.077598 | 0.164 |
|  |  |  | *IL8* | 0.06088 | 0.845 |
|  |  |  | *LTF* | -0.01238 | 0.849 |
|  |  |  | *LYZ* | 0.09545 | 8.61x10^-2^ |
| GSE180238 | Neutrophils (HL60) | *C. trachomatis* | *CXCL1* | 0.05527 | 0.472 |
|  |  |  | *CXCL10 (IP10)* | 7.2164 | 8.19x10^-8^ |
|  |  |  | *IL8* | 1.612433 | 3.27x10^-8^ |
|  |  |  | *LTF* | -0.05034 | 0.706 |
|  |  |  | *LYZ* | -0.21983 | 1.22x10^-2^ |
| GSE180027 | Macrophages | LPS | *CXCL1* | 4.803862 | 7.2x10^-14^ |
|  |  |  | *CXCL10 (IP10)* | 2.052336 | 6.37x10^-3^ |
|  |  |  | *IL8* | 5.416678 | 5.11x10^-4^ |
|  |  |  | *LTF* | -0.21433 | 0.552 |
|  |  |  | *LYZ* | -0.10742 | 0.908 |
